# Supplementary material for: Desmoplastic stroma restricts T cell extravasation and mediates immune exclusion and immunosuppression in solid tumors
Source: Nat Commun. 2023 Aug 22;14:5110. doi: 10.1038/s41467-023-40850-5 (PMC10444764; doi:10.1038/s41467-023-40850-5)
Supplement: Supplementary file 1 — Supplementary Information [file 41467_2023_40850_MOESM1_ESM.pdf]

Supplementary information

Desmoplastic stroma restricts T cell extravasation and mediates immune exclusion and immunosuppression in solid tumors

Zebin Xiao[1], Leslie Todd[1], Li Huang[1], Estela Noguera-Ortega[2], Zhen Lu[1], Lili Huang[3], Meghan Kopp[1], Yue Li[1], Nimisha Pattada[1], Wenqun Zhong[4], Wei Guo[4], John Scholler[5], Maria Liousia[2], Charles-Antoine Assenmacher[6], Carl H. June[5], Steven M. Albelda[2] and Ellen Puré[1,7,*]

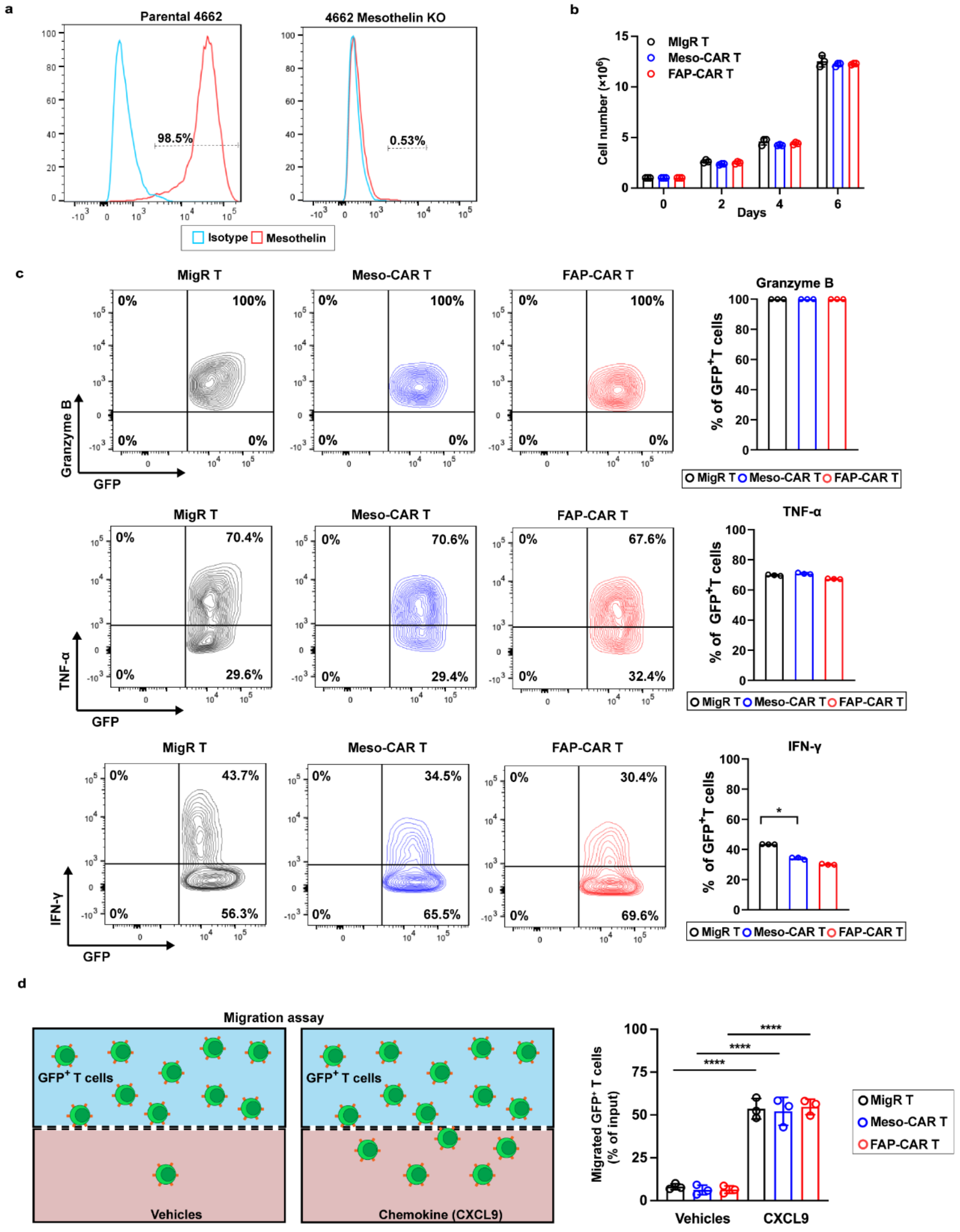

Supplementary Fig. 1 (Related to Fig. 1): Comparable proliferation and functionality of Meso-CAR and FAP-CAR T cells *in vitro*. **(a)** Histogram of mesothelin expression in parental 4662 and 4662 mesothelin KO cell line analyzed by flow cytometry. **(b)** Comparable proliferation of MigR, Meso-CAR T and FAP-CAR T cells induced by anti-CD3/28-beads *in vitro.* **(c)** Representative flow cytometry plots (left) and quantification (right) of CAR T cell expression of GzmB, TNF-α, and IFN-γ 2

days post-activation with anti-CD3/28 beads *in vitro*. **(d)** Migration of MigR, Meso-CAR-T and FAP-CAR T cells in dual chamber wells from upper chamber containing media to lower chamber containing CXCL9 (left) quantified (right). Data indicate mean ± SD (n = 3 independent sample/group). Statistical analysis is performed using one-way ANOVA analysis with Tukey's multiple comparison tests **(b, c and d)**. *$p < 0.05$, and ****$p < 0.0001$. **c.** IFN-γ: MigR vs. Meso-CAR, $p = 0.012$. The p values for remaining comparisons are all < 0.0001. Source data are provided as a Source Data file.

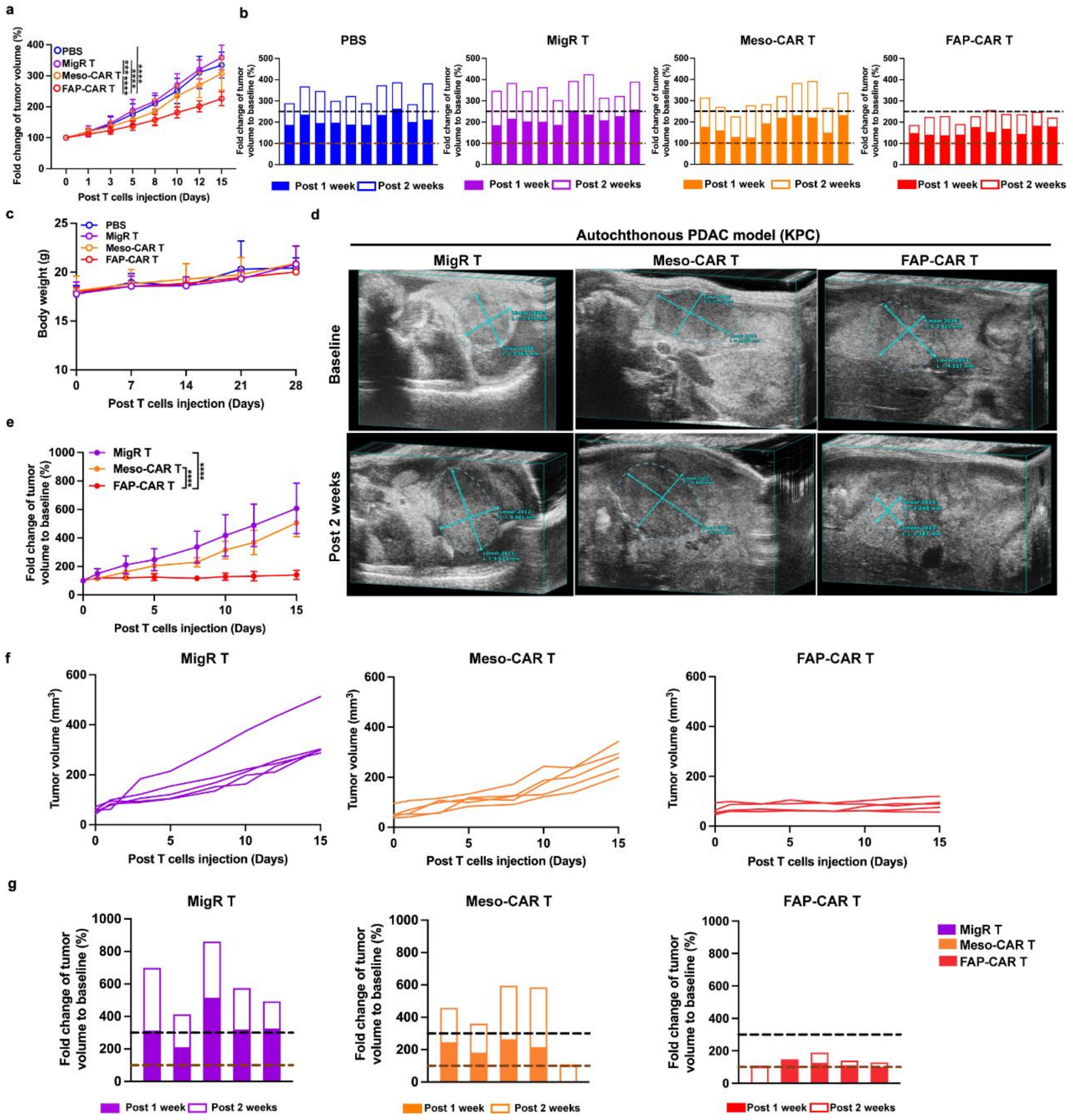

Supplementary Fig. 2 (Related to Fig. 1): Stromal-targeted FAP-CAR T cells inhibited tumor growth more effectively than Meso-CAR T cells in syngeneic transplant models and autochthonous models of PDAC. (a) Average time-dependent fold change in tumor volume in subcutaneous PDAC transplant models with indicated treatments. **(b)** Tumor volume changes for individual transplanted 4662 tumors in syngeneic C57BL/6 mice relative to baseline (just prior to treatment indicated as 100% (brown dashed line) for each individual tumor. Growth above this line indicates tumor progression. The black dashed line indicates each tumor at a volume of 250% compared to baseline. **(c)** Average body weight of 4662 tumor-bearing

C57BL/6 mice with indicated treatments. **(d)** Representative ultrasound images of tumor volume measurement in spontaneous pancreatic tumors in KPC mice following treatment with the indicated CAR T cells. **(e)** Average time-dependent fold change in tumor volume in spontaneous pancreatic tumors in KPC mice following treatment with the indicated CAR T cells. **(f)** Individual tumor growth curves in KPC mice post treatments. **(g)** Tumor volume changes for individual spontaneous pancreatic tumors in KPC mice relative to baseline as described in **b**. Data indicate mean ± SD (n = 10 per group for **a-c**, n = 5 per group for **d-g**) and were compared by one-way ANOVA with Tukey's multiple comparisons test **(a and e)**. ***p < 0.001, and ****p < 0.0001. The p values for all comparisons are < 0.001 or < 0.0001. Source data are provided as a Source Data file.

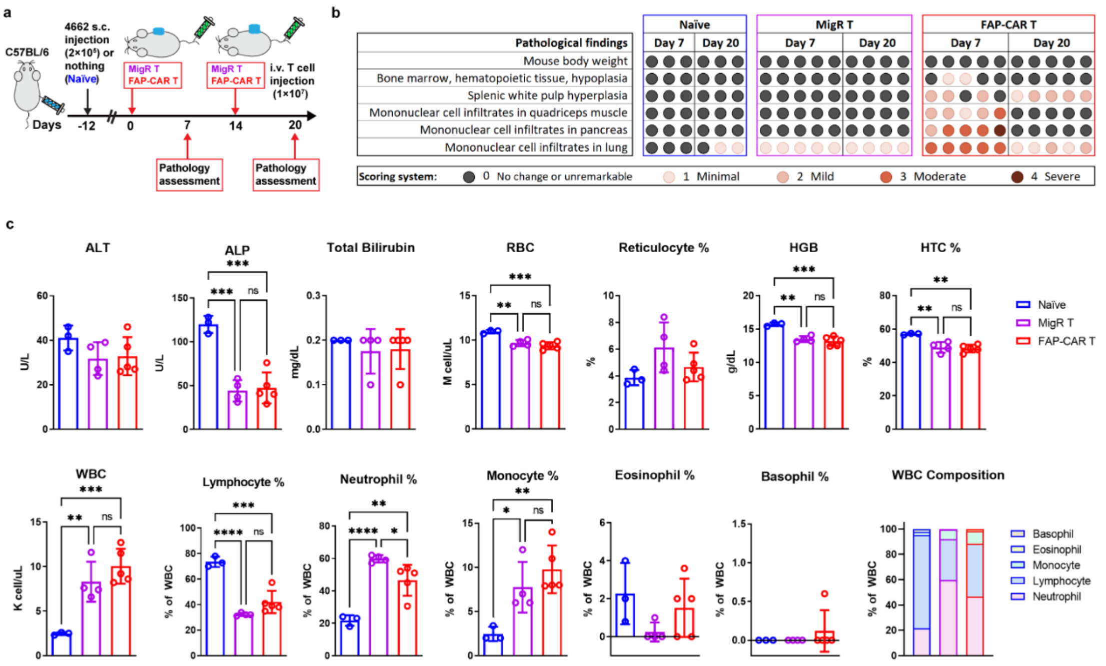

Supplementary Fig. 3 (Related to Fig. 1): FAP-CAR T cells induce reversible pancreatic inflammation. (a) Treatment protocol: 4662 syngeneic PDAC tumor-bearing C57BL/6 mice or naïve mice (non-tumor bearing mice - depicted in blue) treated with one dose or dual doses of MigR control (magenta) or FAP-CAR T cells (red). Pathology assessment was performed 1 week post administration of single or second dose of T cells. **(b)** Pathological assessment of different organs. Colored dots indicate the severity of the lesion or changes in the parameter indicated on the left graded on a semiquantitative scale in 1-point increments. 0 score (gray dot) indicates unremarkable or no change, 1 score (lightest red dot) indicates minimal changes, 2 score (medium red shade dot) indicates mild changes, 3 score (red dot) indicates moderate changes, and 4 score (deep red dot) indicates severe changes. **(c)** Blood biochemistry (upper panel) and white blood cell (WBC) count (bottom panel) was performed at 1 week post second dose of T cells injection. ALT (alanine transaminase), ALP (Alkaline Phosphatase) and total bilirubin (three top left graphs) are indicators of liver function. RBC (Red Blood Count), reticulocyte, HGB (hemoglobin), and HTC (hematocrit), are all parameters that indicate anemia (rest of the top graphs). Data points are mean ± SD (n = 3 for naïve, n = 4 and 5 for MigR control and FAP-CAR T

cells, respectively) and groups were compared using one-way ANOVA analysis with Dukey's multiple comparison tests. n.s. not significant, *p < 0.05, **p < 0.01, ***p < 0.001, ****p < 0.001. **c.** RBC: MigR vs. naïve, p = 0.003. HGB: MigR vs. naïve, p = 0.001. HTC: FAP-CAR vs. naïve, p = 0.002; MigR vs. naïve, p = 0.005. WBC: MigR vs. naïve, p = 0.006. Neutrophils: FAP-CAR vs. MigR, p = 0.038; FAP-CAR vs. naïve, p = 0.002. Monocytes: FAP-CAR vs. naïve, p = 0.007; MigR vs. naïve, p = 0.050. The p values for remaining comparisons are all < 0.001 or < 0.0001. Source data are provided as a Source Data file.

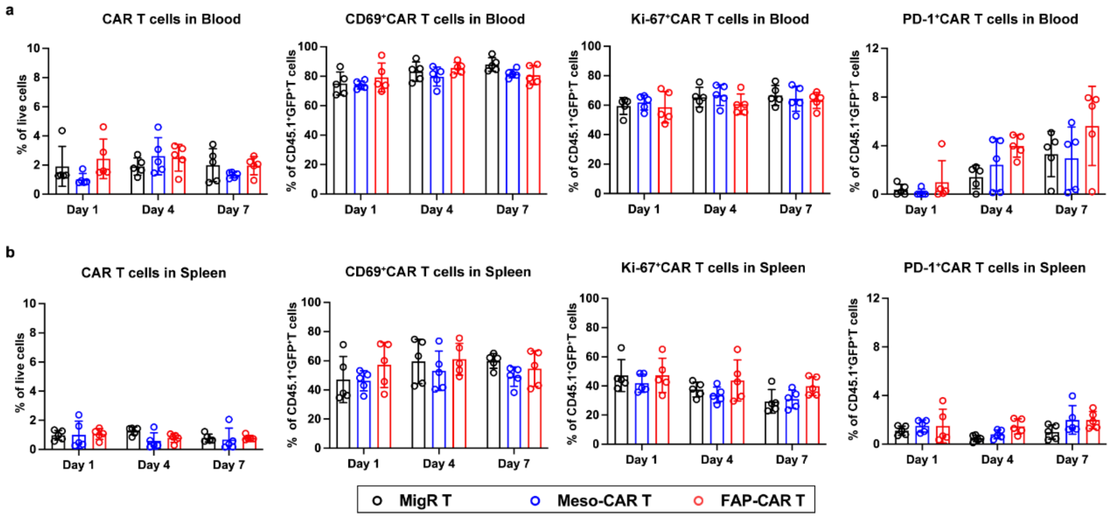

Supplementary Fig. 4 (Related to Fig. 2): FAP-CAR T cells and Meso-CART cells behave comparably in circulation. Quantification and characterization by flow cytometry of CAR T cell expression of CD69, Ki-67 and PD-1 in blood **(a)** and spleen **(b)**. Data indicate mean ± SD (n = 5 per group). P values were determined by one-way ANOVA with Tukey's multiple comparisons test **(a and b)** and no significant differences were found in **a and b**. Source data are provided as a Source Data file.

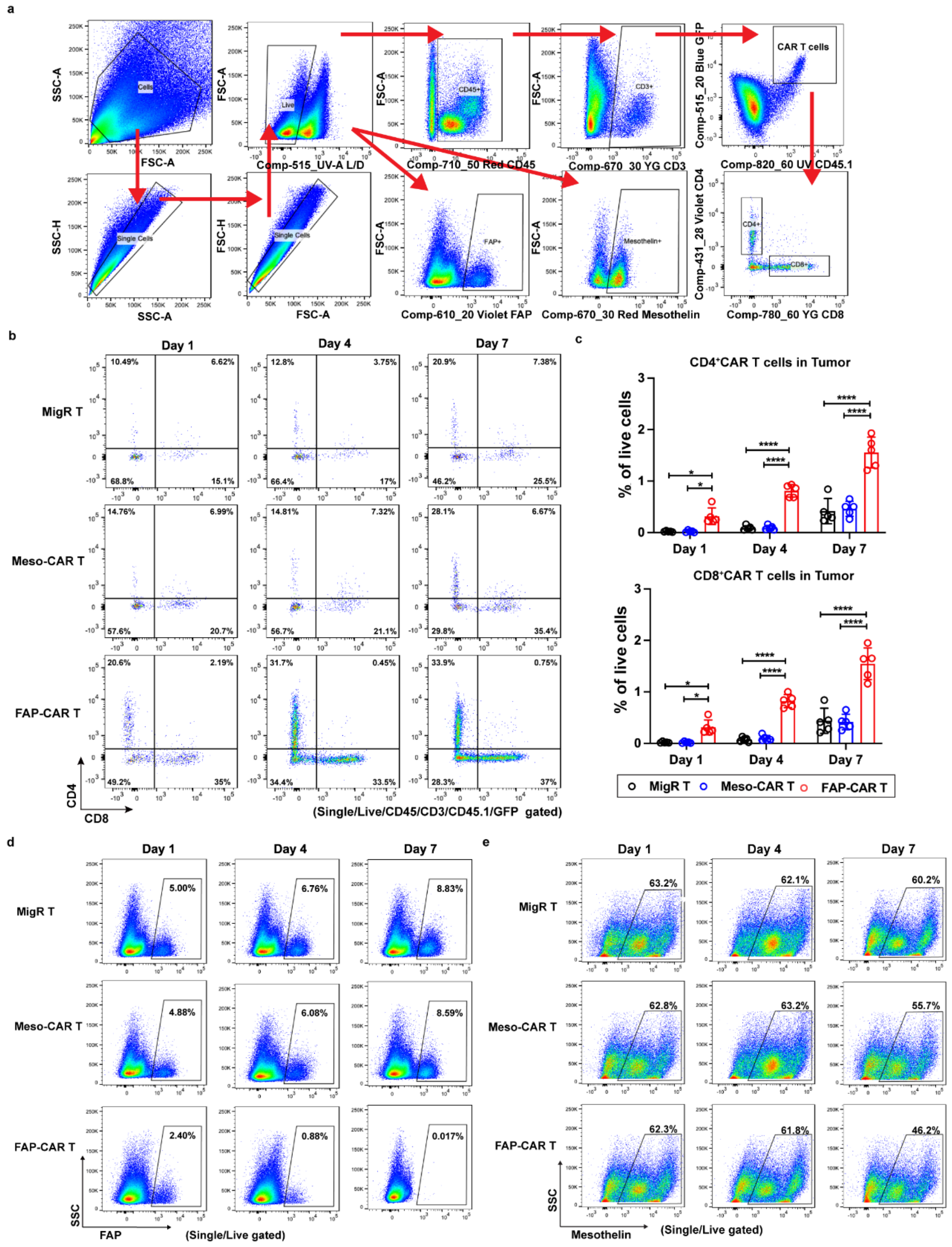

Supplementary Fig. 5 (Related to Fig. 2): FAP-CAR T cells infiltrate and deplete their target cells more effectively than Meso-CAR T cells in PDAC tumors *in vivo*. **(a)** Gating strategy used to analyze flow cytometric data of tumor-infiltrating CAR T cells. **(b)** Representative flow cytometric images and **(c)** quantification of tumor-infiltrating CAR T expression of CD4 (top) and CD8 (bottom). Representative flow

cytometric images of cells with expression of **(d)** FAP (stromal cells) or **(e)** mesothelin (tumor cells) in tumors following treatment with indicated T cells. Data indicates mean ± SD (n = 5 per group). P values were determined by one-way ANOVA with Tukey's multiple comparisons test **(c)**. *p < 0.05, and ****p < 0.0001. **c.** Day 1: CD4 MigR/Meso-CAR vs. FAP-CAR, p = 0.011 and 0.011, respectively. CD8 MigR/Meso-CAR vs. FAP-CAR, p = 0.015 and 0.014, respectively. The p values for remaining comparisons are all < 0.001 or < 0.0001. Source data are provided as a Source Data file.

a

b

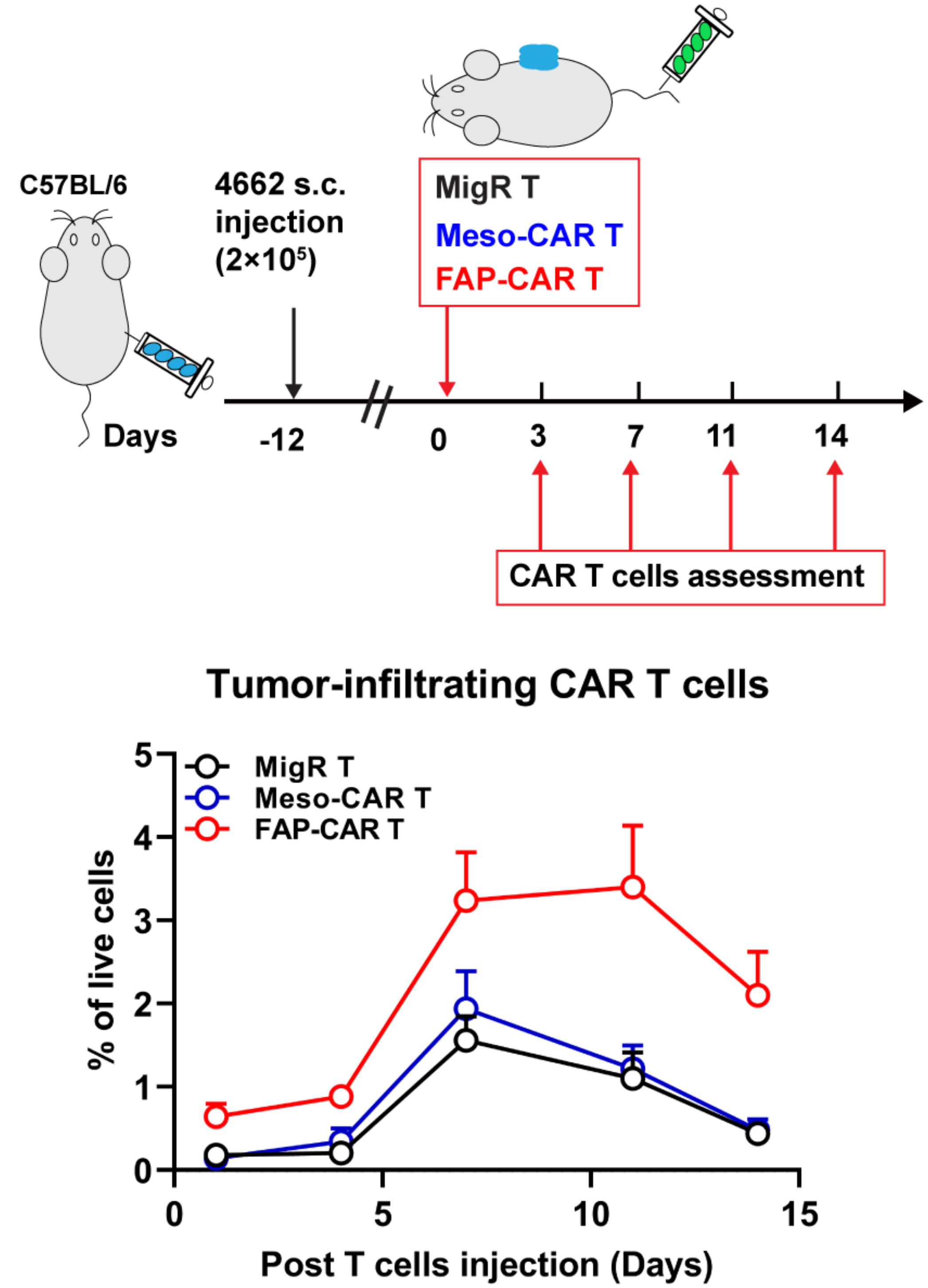

Supplementary Fig. 6: FAP-CAR T cells persist longer than Meso-CAR T cells in PDAC tumors *in vivo*. (a) Treatment protocol: C57BL/6 mice bearing established 4662 tumors were treated with the indicated T cells and tumors were harvested at 3, 7, 11 and 14 days post administration of CAR T cells. **(b)** Quantification of tumor-

infiltrating CAR T cells in the tumors at indicated time points. Data indicates mean ± SD (n = 5 per group). Source data are provided as a Source Data file.

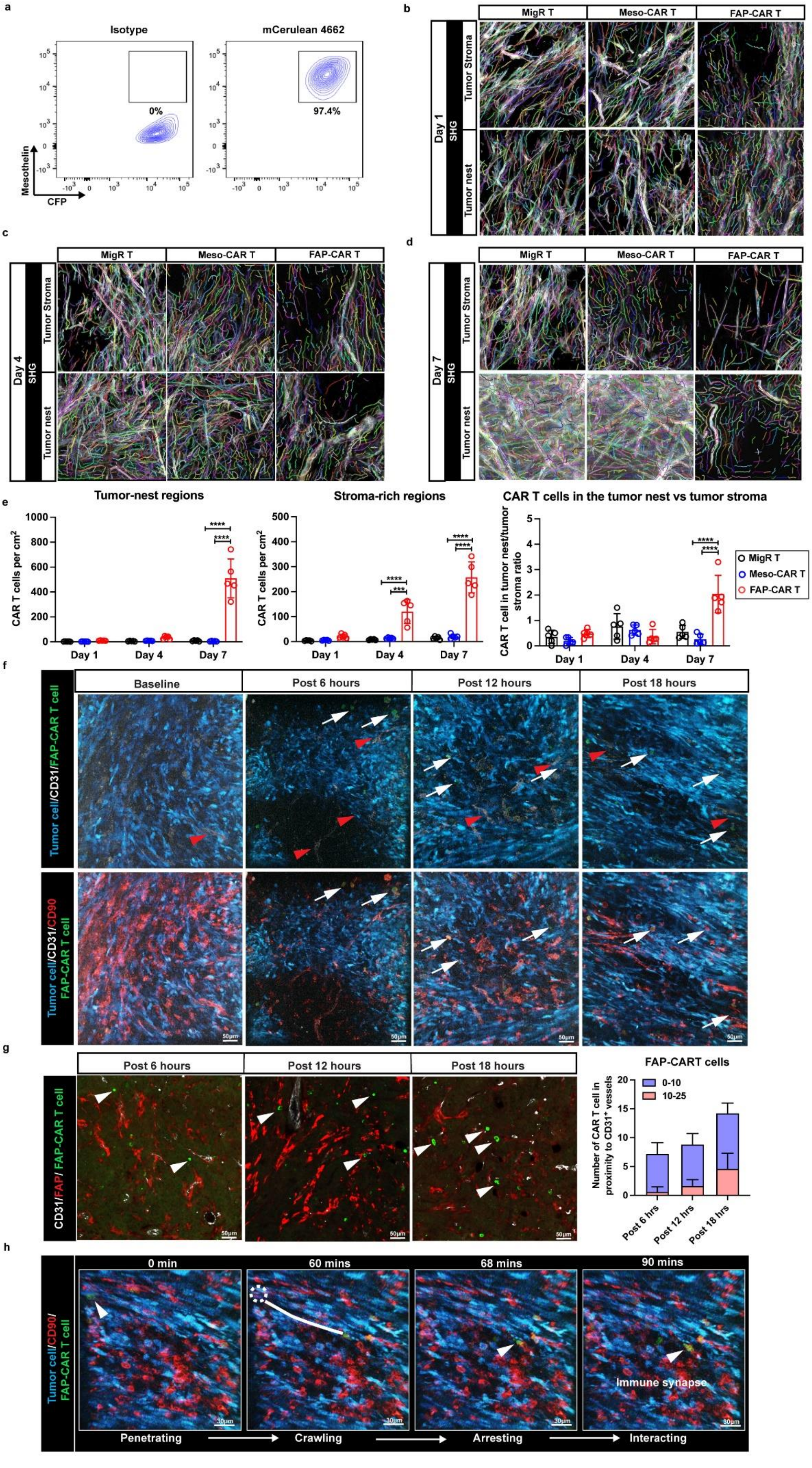
a
Isotype
mCerulean 4662
0%
97.4%
Mesothelin
CFP
b
MigR T
Meso-CAR T
FAP-CAR T
Day 1
SHG
Tumor Stroma
Tumor nest
c
Day 4
d
Day 7
e
Tumor-nest regions
Stroma-rich regions
CAR T cells in the tumor nest vs tumor stroma
CAR T cells per cm^2
CAR T cell in tumor nest/tumor stroma ratio
Day 1
Day 4
Day 7
MigR T
Meso-CAR T
FAP-CAR T
f
Baseline
Post 6 hours
Post 12 hours
Post 18 hours
Tumor cell/CD31/FAP-CAR T cell
Tumor cell/CD31/CD90 FAP-CAR T cell
50μm
g
CD31/FAP/ FAP-CAR T cell
FAP-CART cells
0-10
10-25
Number of CAR T cell in proximity to CD31$^+$ vessels
Post 6 hrs
Post 12 hrs
Post 18 hrs
h
0 min
60 mins
68 mins
90 mins
Tumor cell/CD90/ FAP-CAR T cell
Immune synapse
30μm
Penetrating
Crawling
Arresting
Interacting

Supplementary Fig. 7 (Related to Fig. 3): FAP-CAR T cells overcome the physical barrier and immunosuppressive TME to infiltrate tumor nests. (a) Mesothelin expression in mCerulean 4662 cell line was examined by flow cytometry. **(b-d)** Representative processing and quantification of SHG fiber number in the tumors at stroma-rich and tumor-nest regions at indicated time points post-T cell administration. **(e)** Quantification of the distribution of CAR T cells in the two compartments, including tumor-nest and stroma-rich regions, and the ratio of the number of T cells in tumor nest/in the stroma based on multiplexed IF images. **(f)** Representative static two-photon microscopy images of FAP-CAR T cells in the tumors at indicated time points post-administration, showing the extravasation and penetration of peritumoral regions by FAP-CAR T cells. **(g)** Representative multiplexed IF images (left) and quantification (right) of FAP-CAR T cells (green) located within 10 μm (0-10 μm) or beyond (10-25 μm) the closest $CD31^+$ blood vessel (gray) in the entire area of tumor section. **(h)** A representative time-lapse image of FAP-CAR T cells (green) at 16 hours post-administration. The track of a T cell is shown by the white dotted line and white arrowheads at both ends of the track. *See Supplementary Movie 8*. Similar results were obtained in each of 3 independent experiments **(b-d, f, and h)**. Data points are mean ± SD (n = 5 per group) and groups were compared by one-way ANOVA with Tukey's multiple comparisons test **(e)**. ***p < 0.001, and ****p < 0.0001. The p values for all comparisons are < 0.001 or < 0.0001. Source data are provided as a Source Data file.

Lymphocyte Panel

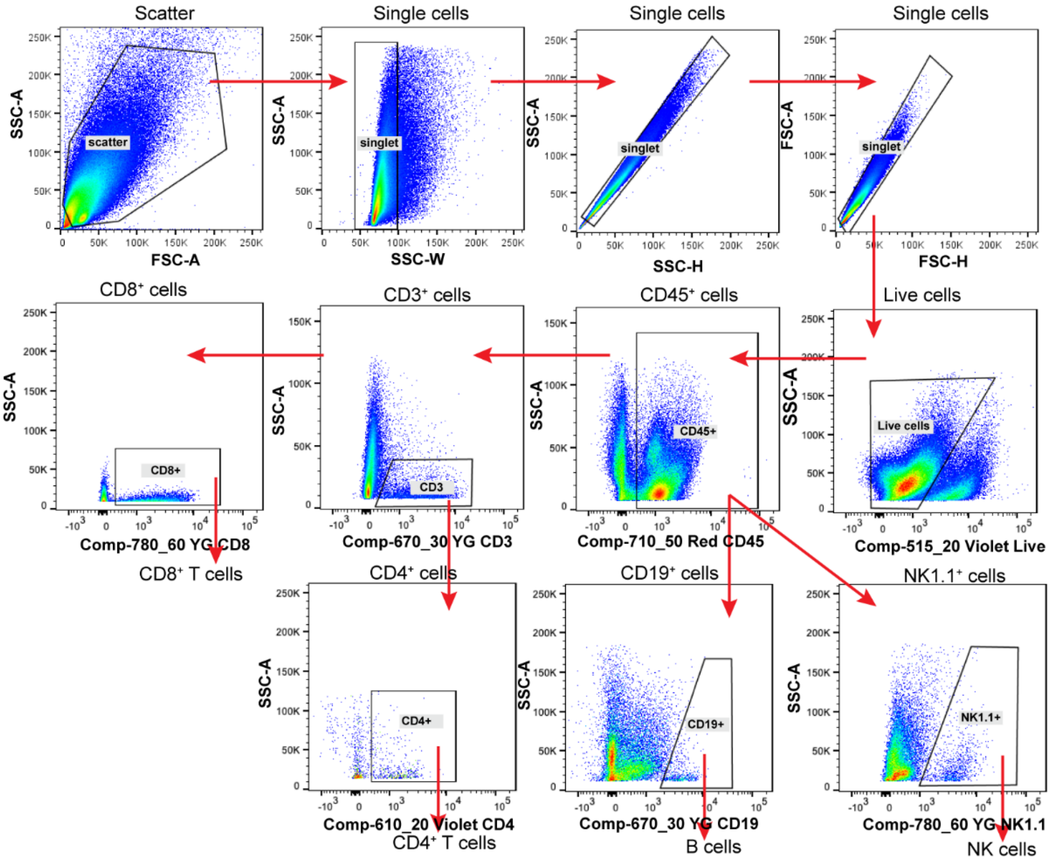

Myeloid cell Panel

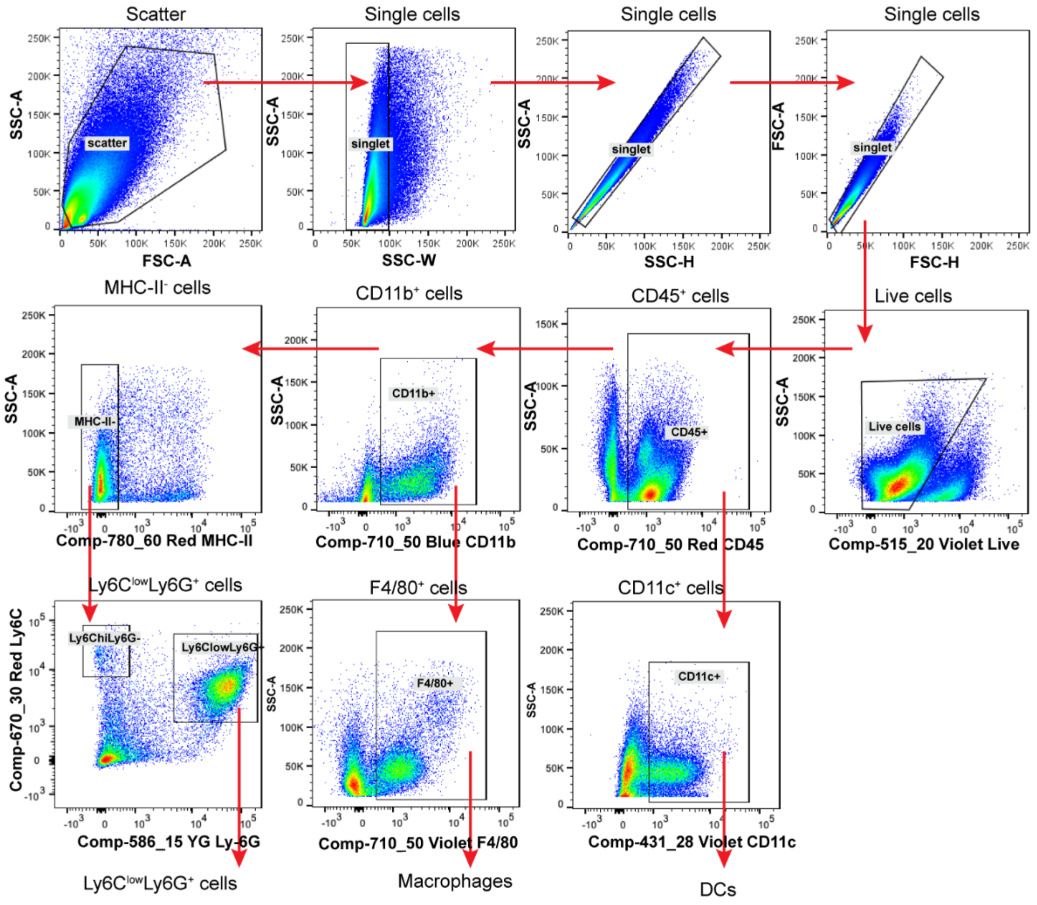

Supplementary Fig. 8 (Related to Fig. 4): Ablation of FAP$^+$ cells alters the stromal landscape, rapidly depletes matrix and enhances T cell infiltration. Gating strategies for profiling immune cells, including T cells, B cells, and myeloid cells depicted in Figure 4b.

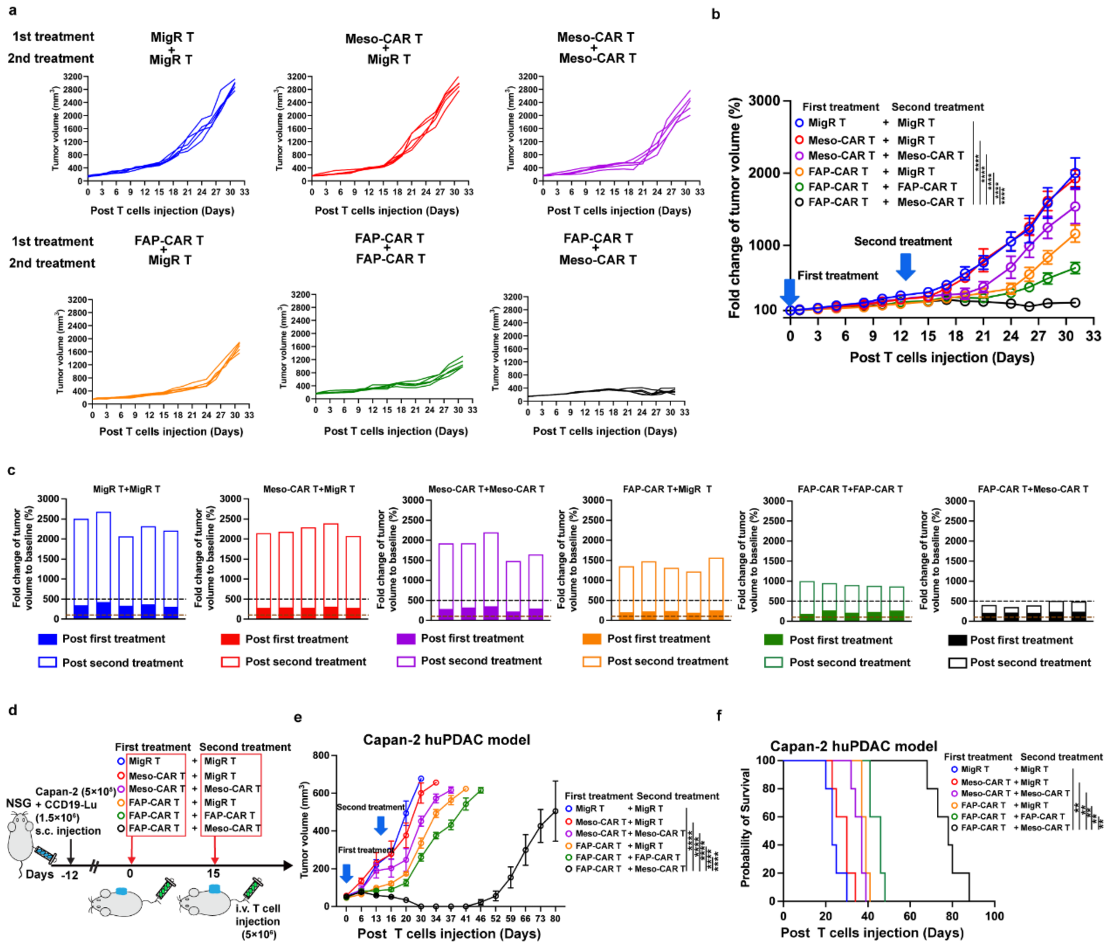

Supplementary Fig. 9 (Related to Fig. 6): Ablation of FAP^{+}-CAFs by FAP-CAR T cells enhances the efficacy of subsequent treatment with TAA Meso-CAR T cells in PDAC tumors. (a) Tumor growth curves of individual 4662 PDAC tumors transplanted into syngeneic mice following treatment with the indicated combination treatments. **(b)** Average fold change in tumor volume over time relative to baseline (just prior to first treatment) in each cohort administered the indicated combination treatments. **(c)** Tumor volume changes of individual 4662 PDAC tumors in mice receiving the indicated combination treatments. **(d)** Treatment protocol: NSG mice bearing established Capan-2 human PDAC tumors were treated with the indicated first dose of T cells and 15 days later treated with the second dose of indicated T cells. **(e)** Average growth curves and **(f)** modified Kaplan-Meier curves of Capan-2 human PDAC bearing NSG mice from each of the indicated treatment groups. Data points are mean ± SD (n = 5 per group) and groups were compared using one-way ANOVA

analysis with Dunnett's multiple comparison tests **(b and e)** or log-rank test **(f)**. *p < 0.05, **p < 0.01, ***p < 0.001, and ****p < 0.0001. **f.** p = 0.002 for all comparisons; the p values for remaining comparisons are all < 0.001 or < 0.0001. Source data are provided as a Source Data file.

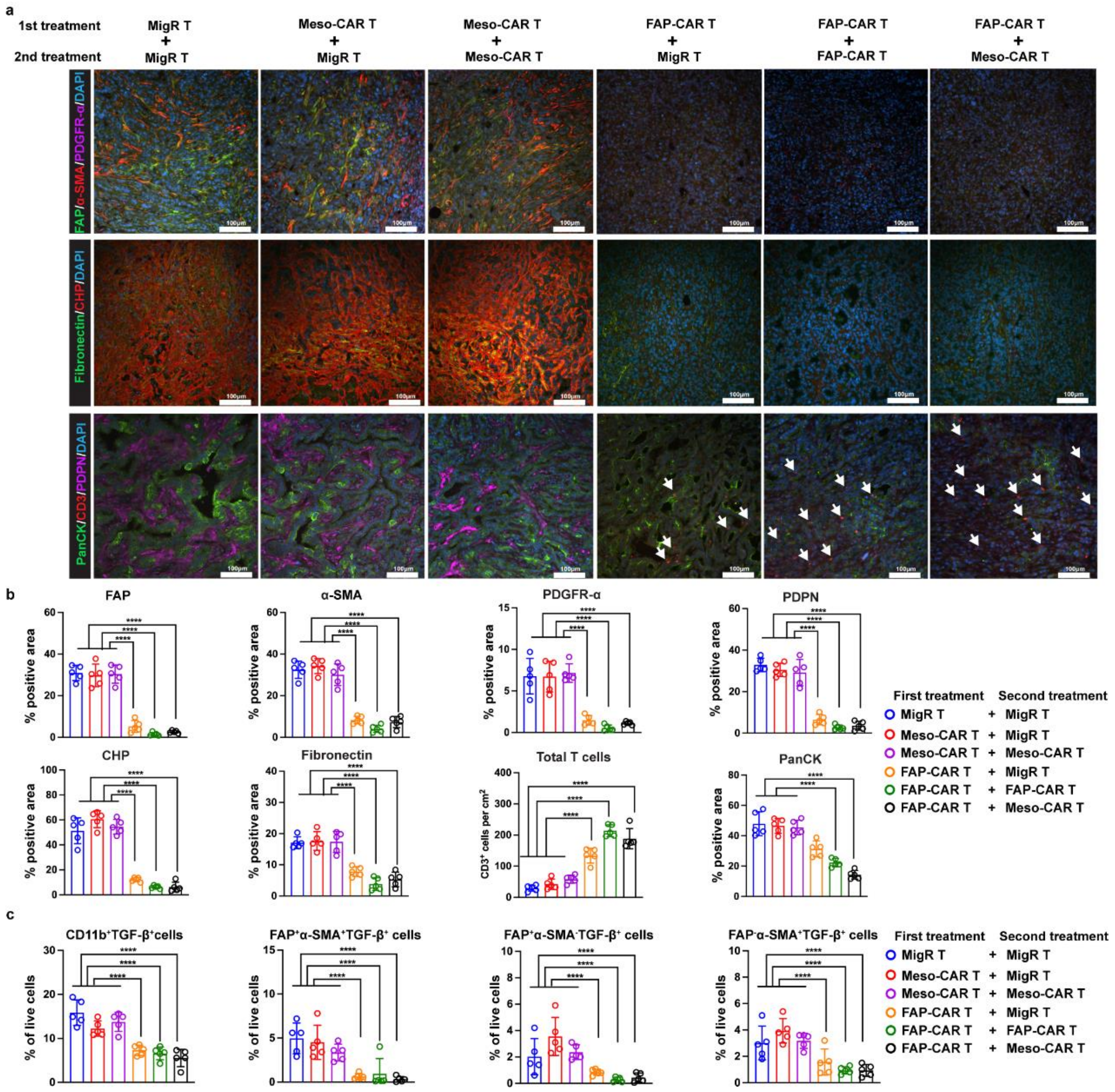

Supplementary Fig. 10 (Related to Fig. 6): Ablation of FAP+-CAFs by FAP-CAR T cells enhances the efficacy of subsequent treatment with TAA Meso-CAR T cells in PDAC tumors. (a) Representative multiplex immunofluorescence images of tumors following indicated combination treatments. Top row, staining of FAP (green), α-SMA (red), PDGFR-α (magenta). Middle row, staining with CHP (red) and FN (green). Bottom row, staining of CD3 (red), Pan-CK (green) and PDPN (magenta). Nuclei were stained with DAPI (blue). Scale bar: 100 μm. Similar results were obtained in each of 3 independent experiments. **(b)** Quantification stromal cells (FAP, α-SMA, PDGFR-α, and PDPN), ECM (CHP and FN), tumor cells (PanCK) and total T cells (CD3) based on multiplexed immunofluorescence of end-point tumors from all mice in each cohort.

(c) Quantification of TGF-β expression in different cell populations, including $CD11b^{+}$, $FAP^{+}\alpha SMA^{+}$, $FAP^{+}\alpha SMA^{-}$ and $\alpha SMA^{+}FAP^{-}$ cells in dissociated end-point tumors from mice receiving indicated combination treatments. Data indicate mean ± SD (n = 5 per group). P values were determined by one-way ANOVA with Tukey's multiple comparisons test **(b and c)**. *p < 0.05, **p < 0.01, ***p < 0.001, and ****p < 0.0001. The p values for all comparisons are all < 0.001 or < 0.0001. Source data are provided as a Source Data file.

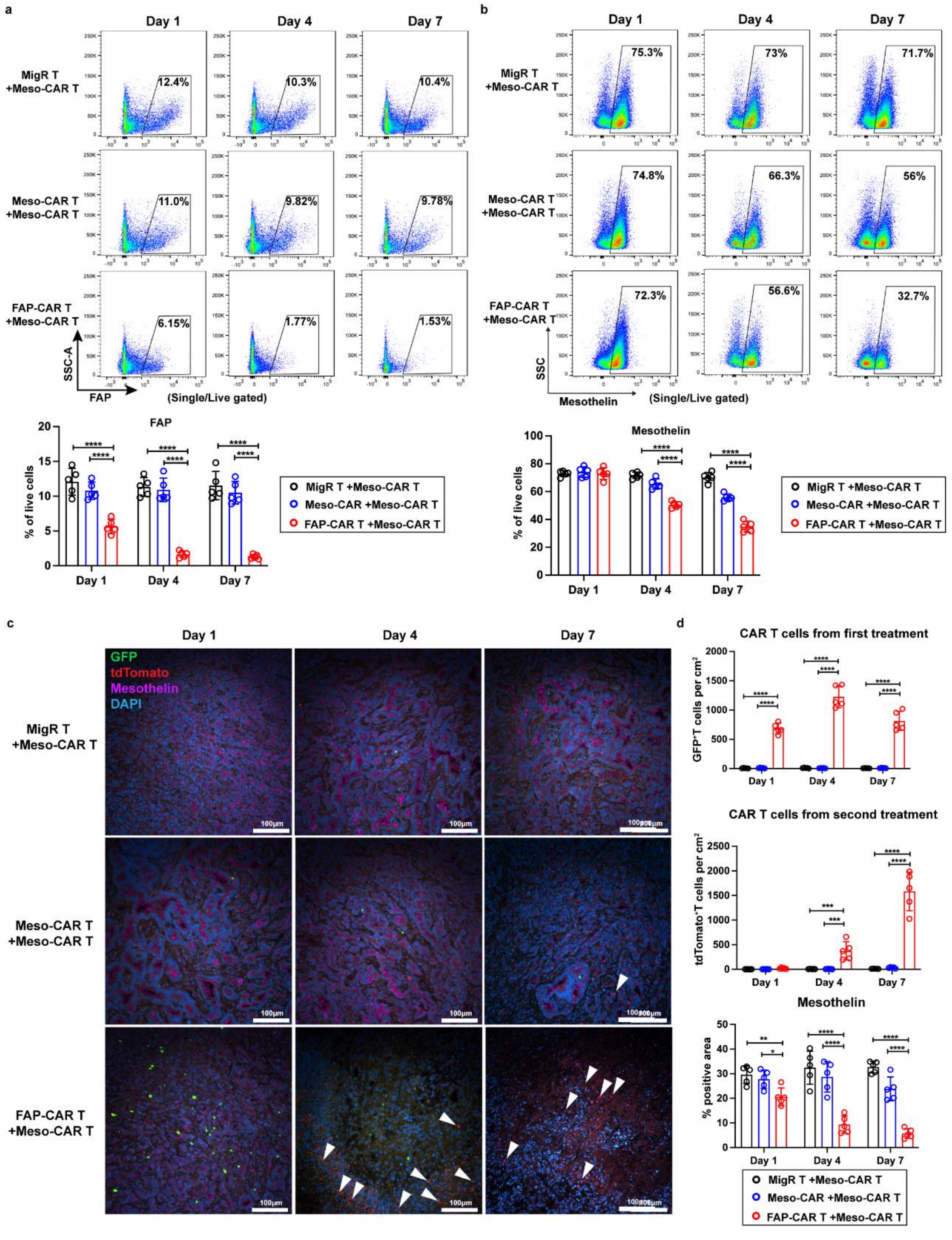

Supplementary Fig. 11 (Related to Fig. 7): Disruption of tumor stroma by FAP-CAR T cells rendered tumors permissive to meso-CAR T cells allowing their infiltration and function within tumor. (a-b) Representative (top) and quantification (bottom) of flow cytometric analysis of **(a)** FAP^{+} and **(b)** mesothelin^{+} targeted cells in tumors post-treatment with indicated combinations. **(c)** Representative multiplex IF images showing CAR T cells from first treatment (GFP^{+}, green) and second treatment

(expressing tdTomato, red) and expression of mesothelin (magenta). Nuclei were stained with DAPI (blue). Scale bar: 100 µm. Three times each experiment was repeated independently with similar results. **(d)** Quantifications of multiplexed immunofluorescence of GFP^+, $tdTomato^+$ and $mesothelin^+$ cells in all tumors from each treatment cohort. Data points are mean ± SD (n = 5 per group) and groups were compared using two-way ANOVA with Tukey's multiple comparisons tests **(a, b and d)**. *p < 0.05, **p < 0.01, ***p < 0.001, and ****p < 0.0001. **d.** middle panel: Day 1, MigR+Meso-CAR vs. FAP-CAR+Meso-CAR, p = 0.006; Meso-CAR+Meso-CAR vs. FAP-CAR+Meso-CAR, p = 0.033. The p values for remaining comparisons are all < 0.001 or < 0.0001. Source data are provided as a Source Data file.

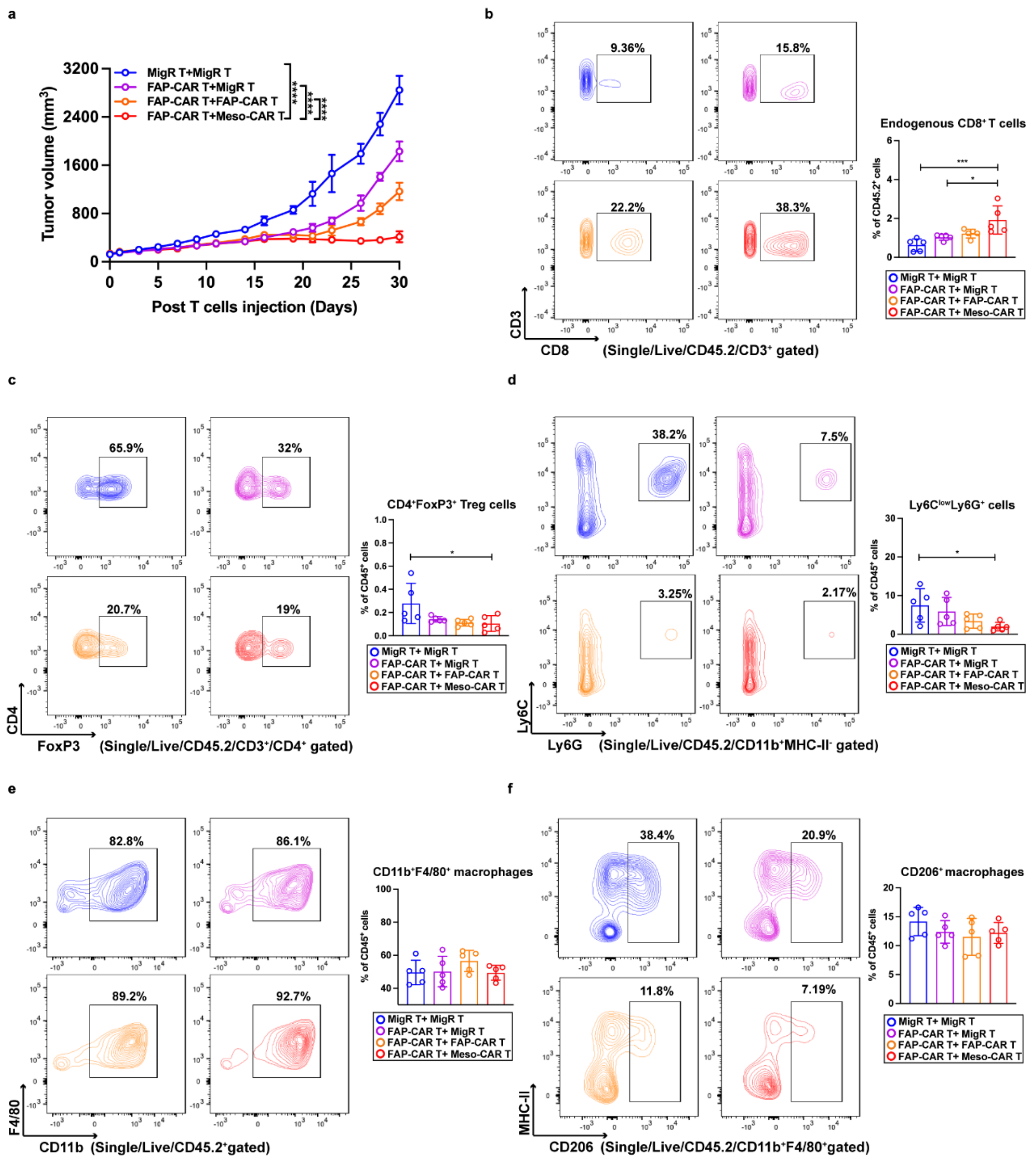

Supplementary Fig. 12 (Related to Fig. 8): Sequential administration of FAP-CAR T cells with Meso-CAR T cells enhances systemic endogenous adaptive anti-tumor immunity in PDAC models. (a) Average growth curves of tumors at end-point for each of the indicated treatment groups. **(b-f)** Representative flow cytometric images (left panels) and quantification (right panels) of CD8$^+$ **(b)**, CD4$^+$FoxP3$^+$ **(c)**, Ly-6C$^+$Ly-6G$^+$ myeloid cells **(d)**, CD11b$^+$F4/80$^+$ macrophages **(e)**, and F4/80$^+$CD206$^+$ macrophages **(f)** in tumors at end-point with indicated combination treatments. Data points are mean ± SD (n = 5 per group) and groups were compared using one-way

ANOVA with Tukey's multiple comparisons test **(a-f)**. *p < 0.05, **p < 0.01, ***p < 0.001, and ****p < 0.0001. **b.** FAP-CAR+MigR vs. FAP-CAR+Meso-CAR, p = 0.019. **c.** MigR+MigR vs. FAP-CAR+Meso-CAR, p =0.049. **d.** MigR+MigR vs. FAP-CAR+Meso-CAR, p = 0.048. The p values for remaining comparisons are all < 0.001 or < 0.0001. Source data are provided as a Source Data file.

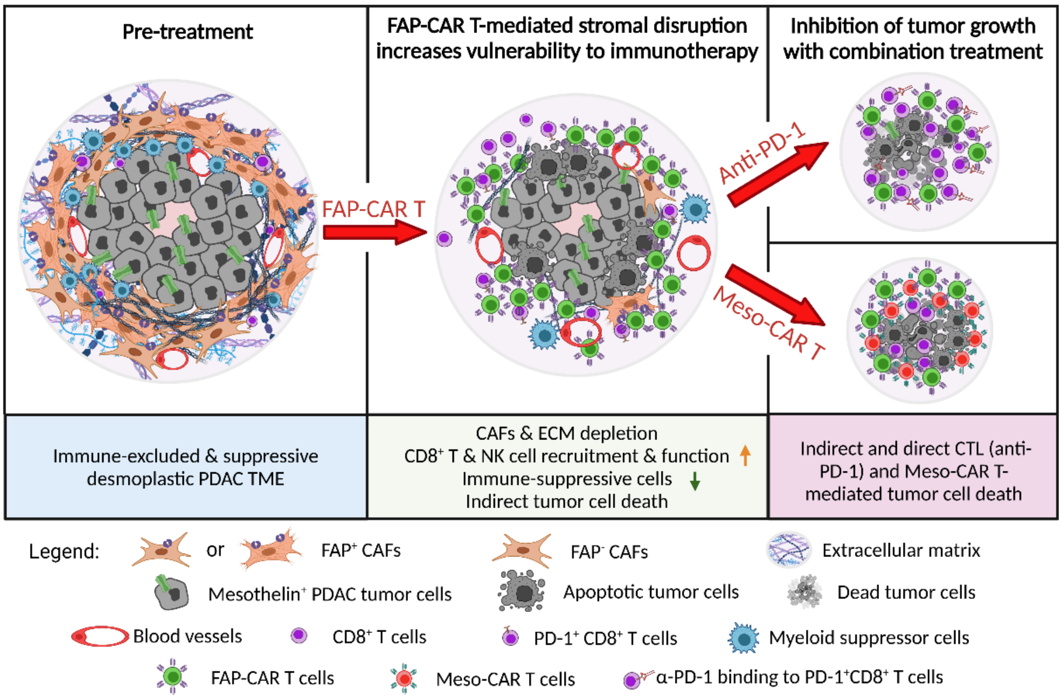

Supplementary Fig. 13: Graphical abstract. Created with BioRender.com.

Supplementary Table 1

REAGENT or RESOURCE	SOURCE	IDENTIFIER
**Antibodies**		
InVivoMAb anti-mouse PD1 (RMP1-14)	BioXCell	Cat#: BE0146; RRID: AB_10949053
CD16/CD32 Monoclonal Antibody (Clone 93)	eBioscience	Cat#: 14-0161-82; RRID: AB_467133
Alexa Fluor® 700 anti-mouse CD45 (Clone QA17A26)	BioLegend	Cat#: 157616; RRID: AB_2890719
Alexa Fluor® 700 anti-mouse CD45.2 (Clone 104)	BioLegend	Cat#: 109822; RRID: AB_493731
Brilliant Violet 605™ anti-mouse CD45.1 Antibody (Clone A20)	BioLegend	Cat#: 110738; RRID: AB_2562565
APC/Cyanine7 anti-mouse CD45.1 Antibody (Clone A20)	BioLegend	Cat#: 110716; RRID: AB_313505
BUV805 Mouse Anti-Mouse CD45.1 (Clone A20)	BD Biosciences	Cat#: 612900; RRID: AB_2739008
PE/Cyanine5 anti-mouse CD3ε (Clone 145-2C11)	BioLegend	Cat#: 100310; RRID: AB_312675
APC/Cyanine7 anti-mouse CD4 Antibody (Clone GK1.5)	BioLegend	Cat#: 100414; RRID: AB_312699
BUV805 Rat Anti-Mouse CD4 (Clone GK1.5)	BD Biosciences	Cat#: 612900; RRID: AB_2739008
Brilliant Violet 421™ anti-mouse CD4 Antibody (Clone GK1.5)	BioLegend	Cat#: 100438; RRID: AB_11203718
PE-Cyanine7 CD8a Monoclonal Antibody (Clone 53-6.7)	eBioscience	Cat#: 25-0081-82; RRID: AB_469584
Brilliant Violet 785™ anti-mouse CD279 (PD-1) Antibody (Clone 29F.1A12)	BioLegend	Cat#: 135225; RRID: AB_2563680
Brilliant Violet 650™ anti-mouse/human Ki-67 Antibody (Clone 11F6)	BioLegend	Cat#: 151215; RRID: AB_2876504
BUV395 Mouse Anti-Mouse CD366 (TIM-3) (Clone 5D12)	BD Biosciences	Cat#: 747620; RRID: AB_2744186
PE anti-mouse CD69 Antibody (Clone H1.2F3)	BioLegend	Cat#: 104508; RRID: AB_313111
Brilliant Violet 421™ anti-mouse LAP (TGF-β1) Antibody (Clone TW7-16B4)	BioLegend	Cat#: 141408; RRID: AB_2650898
PerCP/Cyanine5.5 anti-mouse LAP (TGF-β1) Antibody (Clone TW7-16B4)	BioLegend	Cat#: 141410; RRID: AB_2561592
Alexa Fluor® 647 anti-mouse FOXP3 Antibody (Clone MF-14)	BioLegend	Cat#: 126408; RRID: AB_1089115
PerCP-Cy™5.5 Rat Anti-CD11b (Clone M1/70)	BD Biosciences	Cat#: 550993; RRID: AB_394002
Brilliant Violet 605™ anti-mouse Ly-6C Antibody (Clone HK1.4)	BioLegend	Cat#: 128036; RRID: AB_2562353
APC anti-mouse Ly-6C Antibody (Clone HK1.4)	BioLegend	Cat#: 128016; RRID: AB_1732076
APC/Cyanine7 anti-mouse I-A/I-E Antibody (Clone M5/114.15.2)	BioLegend	Cat#: 107628; RRID: AB_2069377
PE/Cyanine5 anti-mouse I-A/I-E Antibody (Clone M5/114.15.2)	BioLegend	Cat#: 107612; RRID: AB_313327
PE anti-mouse Ly-6G Antibody (Clone 1A8)	BioLegend	Cat#: 127608; RRID: AB_1186099
BUV395 Rat Anti-Mouse Ly-6G (Clone 1A8)	BD Biosciences	Cat#: 563978; RRID: AB_2716852
BUV805 Hamster Anti-Mouse CD11c (Clone HL3)	BD Biosciences	Cat#: 749090; RRID: AB_2873482
BV421 Hamster Anti-Mouse CD11c (Clone HL3)	BD Biosciences	Cat#: 562782; RRID: AB_2737789

Brilliant Violet 650™ anti-mouse CD11c Antibody (Clone N418)	BioLegend	Cat#: 117339; RRID: AB_2562414
Brilliant Violet 605™ anti-mouse CD103 Antibody (Clone 2E7)	BioLegend	Cat#: 121433; RRID: AB_2629724
Brilliant Violet 650™ anti-mouse CD206 (MMR) Antibody (Clone C068C2)	BioLegend	Cat#: 141723; RRID: AB_2562445
BV711 Rat Anti-Mouse F4/80 (Clone T45-2342)	BD Biosciences	Cat#: 565612; RRID: AB_2734769
PE/Cyanine5 anti-mouse CD19 Antibody (Clone 6D5)	BioLegend	Cat#: 115510; RRID: AB_313645
PE/Cyanine7 anti-mouse NK-1.1 Antibody (Clone S17016D)	BioLegend	Cat#: 156514; RRID: AB_2888852
APC/Cyanine7 anti-mouse CD326 (Ep-CAM) Antibody (Clone G8.8)	BioLegend	Cat#: 118218; RRID: AB_2098648
Alexa Fluor® 647 anti-mouse CD326 (Ep-CAM) Antibody (Clone G8.8)	BioLegend	Cat#: 118212; RRID: AB_1134101
PE/Cyanine7 anti-mouse CD326 (Ep-CAM) Antibody (Clone G8.8)	BioLegend	Cat#: 118216; RRID: AB_1236471
Brilliant Violet 785™ anti-mouse CD90.2 (Thy-1.2) Antibody (Clone 30-H12)	BioLegend	Cat#: 105331; RRID: AB_2562900
PE anti-mouse CD90.2 (Thy-1.2) Antibody (Clone 30-H12)	BioLegend	Cat#: 105308; RRID: AB_313179
Alexa Fluor® 647 anti-mouse CD90.2 (Thy1.2) Antibody (Clone 30-H12)	BioLegend	Cat#: 105318; RRID: AB_492888
Alexa Fluor® 647 anti-mouse CD31 Antibody (Clone 390)	BioLegend	Cat#: 102416; RRID: AB_493410
APC AffiniPure F(ab')$_2$ Fragment Goat Anti-Mouse IgG, F(ab')$_2$ fragment specific	Jackson ImmunoResearch	Cat#: 115-136-072; RRID: AB_2338649
APC AffiniPure F(ab')$_2$ Fragment Goat Anti-Human IgG, F(ab')$_2$ fragment specific	Jackson ImmunoResearch	Cat#: 109-136-097; RRID: AB_2337692
Mouse monoclonal Anti-Actin, α-Smooth Muscle - Cy3™ antibody (Clone 1A4)	Sigma-Aldrich	Cat#: C6198; RRID: AB_476856
PerCP/Cyanine5.5 anti-human/mouse Granzyme B Recombinant Antibody (Clone QA16A02)	BioLegend	Cat#: 372212; RRID: AB_2728379
PE anti-human/mouse Granzyme B Recombinant Antibody (Clone QA16A02)	BioLegend	Cat#: 372208; RRID: AB_2687032
PE anti-mouse IFN-γ Antibody (Clone XMG1.2)	BioLegend	Cat#: 505808; RRID: AB_315402
Brilliant Violet 711™ anti-mouse IFN-γ Antibody (Clone XMG1.2)	BioLegend	Cat#: 505836; RRID: AB_2650928
APC anti-mouse TNF-α Antibody (Clone MP6-XT22)	BioLegend	Cat#: 506308; RRID: AB_315429
Brilliant Violet 650™ anti-mouse TNF-α Antibody (Clone MP6-XT22)	BioLegend	Cat#: 506333; RRID: AB_2562450
Anti-mouse FAP (Clone 73.3), Biotinylated	Wang LC, et al. Cancer Immunol Res[1]	N/A
Alexa Fluor™ 488 Pan Cytokeratin Monoclonal Antibody (Clone AE1/AE3)	eBioscience	Cat#: 53-9003-82; RRID: AB_1834350
Recombinant Anti-Fibroblast activation protein, alpha antibody (Clone EPR20021)	Abcam	Cat#: ab207178; RRID: AB_2864720
Goat polyclonal Anti-GFP antibody	Abcam	Cat#: ab6673; RRID: AB_305643

Rabbit polyclonal Anti-CD3 antibody	Abcam	Cat#: ab5690; RRID: AB_305055
Recombinant Anti-CD8 alpha antibody (EPR21769)	Abcam	Cat#: ab217344; RRID: AB_2890649
Recombinant Anti-CD4 antibody (EPR19514)	Abcam	Cat#: ab183685; RRID: AB_2686917
Recombinant Anti-FOXP3 antibody (EPR22102-37)	Abcam	Cat#: ab215206; RRID: AB_2860568
Recombinant Anti-F4/80 antibody (SP115)	Abcam	Cat#: ab111101; RRID: AB_10859466
Recombinant Anti-CD103 antibody (EPR22590-27)	Abcam	Cat#: ab224202; RRID: AB_2891141
Anti-Ly6g+Ly6c (Gr-1) antibody (RB6-8C5)	Abcam	Cat#: ab25377; RRID: AB_470492
Recombinant Anti-Ki67 antibody (SP6)	Abcam	Cat#: ab16667; RRID: AB_302459
Recombinant Anti-Cytokeratin 19 antibody (EP1580Y)	Abcam	Cat#: ab52625; RRID: AB_2281020
Recombinant Anti-Mesothelin antibody (EPR17823-52)	Abcam	Cat#: ab187063
Mesothelin Monoclonal Antibody (MSLN, Clone 2131)	NeoBiotechologies	Cat#: 10232-MSM1-P1
Monoclonal Rat anti-Mouse MSLN/Mesothelin Antibody (clone B35)	LSBio	Cat#: LS-C179484-100
TGF beta 1 Polyclonal Antibody	Bioss	Cat#: BS-0086R
Mouse/Rat CD31/PECAM-1 Antibody	R&D Systems	Cat#: AF3628; RRID: AB_2161028
Mouse PDGFR alpha Antibody	R&D Systems	Cat#: AF1062; RRID: AB_2236897
Mouse Podoplanin Antibody	R&D Systems	Cat#: AF3244; RRID: AB_2268062
Mouse/Rat IFN-gamma Antibody	R&D Systems	Cat#: AF-585-NA
Mouse MMR/CD206 Antibody	R&D Systems	Cat#: AF2535; RRID: AB_2063012
Mouse PD-1 Antibody	R&D Systems	Cat#: AF1021; RRID: AB_354541
Rabbit Polyclonal Anti-RFP Antibody	Rockland	Cat#: 600-401-379; RRID: AB_2209751
Collagen Hybridizing Peptide, Biotin Conjugate (B-CHP)	3Helix	Cat#: BIO300
Rabbit Polyclonal Anti-Fibronectin antibody	Sigma-Aldrich	Cat#: F3648; RRID: AB_476976
Rabbit Cleaved Caspase-3 (Asp175) (5A1E) mAb	Cell Signaling Technology	Cat#: 9664; RRID: AB_2070042
Alexa Fluor™ Plus 488 Donkey anti-Rabbit IgG (H+L) Highly Cross-Adsorbed Secondary Antibody	Invitrogen	Cat#: A32790; RRID: AB_2762833
Alexa Fluor™ Plus 555 Donkey anti-Rabbit IgG (H+L) Highly Cross-Adsorbed Secondary Antibody	Invitrogen	Cat#: A32794; RRID: AB_2762834
Alexa Fluor™ Plus 647 Donkey anti-Rabbit IgG (H+L) Highly Cross-Adsorbed Secondary Antibody	Invitrogen	Cat#: A32795; RRID: AB_2762835
Alexa Fluor™ Plus 488 Donkey anti-Goat IgG (H+L) Highly Cross-Adsorbed Secondary Antibody	Invitrogen	Cat#: A32814; RRID: AB_2762838
Alexa Fluor™ Plus 555 Donkey anti-Goat IgG (H+L) Highly Cross-Adsorbed Secondary Antibody	Invitrogen	Cat#: A32816; RRID: AB_2762839
Alexa Fluor™ Plus 647 Donkey anti-Goat IgG (H+L) Highly Cross-Adsorbed Secondary Antibody	Invitrogen	Cat#: A32849; RRID: AB_2762840

DyLight™ 488 Donkey anti-Rat IgG (H+L) Cross-Adsorbed Secondary Antibody	Invitrogen	Cat#: SA5-10026; RRID: AB_2556606
DyLight™ 550 Donkey anti-Rat IgG (H+L) Cross-Adsorbed Secondary Antibody	Invitrogen	Cat#: SA5-10027; RRID: AB_2556607
DyLight™ 650 Donkey anti-Rat IgG (H+L) Cross-Adsorbed Secondary Antibody	Invitrogen	Cat#: SA5-10029; RRID: AB_2556609
DyLight™ 488 Donkey anti-Mouse IgG (H+L) Cross-Adsorbed Secondary Antibody	Invitrogen	Cat#: SA5-10166; RRID: AB_2556746
DyLight™ 550 Donkey anti-Mouse IgG (H+L) Cross-Adsorbed Secondary Antibody	Invitrogen	Cat#: SA5-10167; RRID: AB_2556747
DyLight™ 560 Donkey anti-Mouse IgG (H+L) Cross-Adsorbed Secondary Antibody	Invitrogen	Cat#: SA5-10169; RRID: AB_2556749
**Chemicals, peptides, and recombinant proteins**		
Dynabeads Mouse T-Activator CD3/CD28 for T-Cell Expansion and Activation	Gibco	Cat#: 11452D
Lipofectamine™ 2000 Transfection Reagent	Invitrogen	Cat#: 52887
Lysing Buffer (10x)	BD Biosciences	Cat#: 555899
4',6-diamidino-2-phenylindole (DAPI)	Roche	Cat#: 10236276001
LIVE/DEAD™ Fixable Aqua Dead Cell Stain Kit, for 405 nm excitation	Invitrogen	Cat#: L34957
Alexa Fluor™ 488 Tyramide SuperBoost™ Kit, goat anti-rabbit IgG	Invitrogen	Cat#: B40943
Alexa Fluor™ 594 Tyramide SuperBoost™ Kit, goat anti-rabbit IgG	Invitrogen	Cat#: B40925
Alexa Fluor™ 647 Tyramide SuperBoost™ Kit, goat anti-rabbit IgG	Invitrogen	Cat#: B40926
ProLong™ Diamond Antifade Mountant with DAPI	Invitrogen	Cat#: P36971
Streptavidin, Alexa Fluor™ 555	Invitrogen	Cat#: S21381
BD Stain Buffer	BD Biosciences	Cat#: 554657
Cell Activation Cocktail (with Brefeldin A)	BioLegend	Cat#: 423304
Fixation/Permeabilization Solution Kit with BD GolgiStop™	BD Biosciences	Cat#: 554715
Foxp3/Transcription Factor Staining Buffer Set	eBioscience	Cat#: 00-5523-00
Brilliant Violet 605™ Streptavidin	BioLegend	Cat#: 405229
PE/Dazzle™ 594 Streptavidin	BioLegend	Cat#: 405248
PE/Cyanine7 Streptavidin	BioLegend	Cat#: 405206
Biochemical Corporation Collagenase, Type 2	Worthington	Cat#: LS004176
Interleukin-2, mouse (mIL-2) recombinant (E. coli)	Roche	Cat#: 11271164001
2-Mercaptoethanol (100X)	Sigma-Aldrich	Cat#: ES-007-E
RetroNectin® Recombinant Human Fibronectin Fragment	Takara	Cat#: T100B
DNase I recombinant, RNase-free	Roche	Cat#: 04716728001
Citrate Buffer, pH 6.0, 10x	Sigma-Aldrich	Cat#: C9999

Triton™ X-100	Sigma-Aldrich	Cat#: X100
Recombinant Murine CXCL9	Peprotech	Cat#: 250-18
Agarose, low gelling temperature Type VII-A	Sigma-Aldrich	Cat#: A0701
Bovine Serum Albumin-Fraction V	Rockland	Cat#: BSA-50
Fetal Bovine Serum	GemCell	Cat#: 100-500
Penicillin-Streptomycin	Gibco	Cat#: 15140-122
L-Glutamine	Corning	Cat#: 25-005-CI
Sodium Pyruvate	Corning	Cat#: 25-000-CI
Cell Dissociation Buffer Enzyme-Free PBS-based	Gibco	Cat#: 13151-014
RPMI 1640 Medium, no phenol red	Gibco	Cat#: 11835030
Prefer fixative solution	ANATECH	Cat#: 410
**Critical commercial assays**		
EasySep Mouse T Cell Isolation Kit	STEMCELL Technologies	Cat#: 19851
Transwell Chambers	Millipore	Cat#: CLS3422-48EA
Millicell Cell Culture Insert, 30 mm, hydrophilic PTFE, 0.4 µm	Millipore	Cat#: PICM03050
MycoAlert® Mycoplasma Detection Kit	Lonza	Cat#: LT07-318
Luciferase Assay System	Promega	Cat#: E1500
CellTiter 96® AQueous Non-Radioactive Cell Proliferation Assay (MTS)	Promega	Cat#: G5421
Mouse IFN-gamma Quantikine ELISA Kit	R&D Systems	Cat#: MIF00
Slice Anchors, 19.7 diameter, 2 mm spacing threads	Warner Instruments	Cat#: 64-1415
**Experimental models: Cell lines**		
Human: Phoenix-ECO	ATCC	Cat#: CRL-3214™; RRID: CVCL_H717
Human: 293T/17 [HEK 293T/17]	ATCC	Cat#: CRL-11268™; RRID: CVCL_1926
Human: CCD-19Lu	ATCC	Cat# CCL-210™; RRID: CVCL_2382
Human: AsPC-1	ATCC	Cat# CRL-1682™; RRID: CVCL_0152
Mouse: 4662 PDAC cells	Lo A, et al. Cancer Res[2]	N/A
Mouse: mCerulean 4662 PDAC cells	This paper	N/A
Mouse: 4662 mesothelin KO PDAC cells	This paper	N/A
Mouse: 3T3.mFAP.GFP/Luciferase	Wang LC, et al. Cancer Immunol Res[1]	N/A
Mouse: 3T3.GFP/Luciferase	Wang LC, et al. Cancer Immunol Res[1]	N/A
**Experimental models: Organisms/strains**		
Mouse: WT C57BL/6J	Jackson Laboratory	Strain Code: 000664
Mouse: B6.SJL-*Ptprc*^{a} *Pepc*^{b}/BoyJ (B6 CD45.1)	Jackson Laboratory	Strain Code: 002014
Mouse: NOD.Cg-*Prkdc*^{scid} *Il2rg*^{tm1Wjl}/SzJ (NSG)	Jackson Laboratory	Strain Code: 005557
Mouse: B6.129(Cg)-*Gt(ROSA)26Sor*$^{tm4(ACTB-tdTomato,-EGFP)Luo}$/J (mT/mG)	Jackson Laboratory	Strain Code: 007676

Mouse: *$Kras^{G12D}$;$Trp53^{R172H}$;Pdx-1-Cre* (KPC)	Lo A, et al. Cancer Res 2015	N/A
**Recombinant DNA**		
MSGV-moMeso A03-3 MuBBz	Watanabe K, et al. JCI Insight[3]	N/A
MSGV-moMeso-T2A-GFP (A03-3)	Watanabe K, et al. JCI Insight[3]	N/A
migR1.huMeso-CD8-41BB-CD3z-IRES-GFP (M11)	Klampatsa A. et al. Mol Ther Oncolytics[4]	N/A
migR1.4G5(L2HG)-CD8-41BB-CD3z-IRES-GFP	Lee IK, et al. Clin Cancer Res[5]	N/A
pMSCVpuro-mCerulean3	Addgene	Cat#: 96936
lentiCRISPRv2 hygro	Addgene	Cat#: 98291
**Software and algorithms**		
GraphPad PRISM 9	GraphPad	RRID:SCR_002798
FlowJo (version 10.7.2)	FlowJo LLC.	RRID:SCR_008520
FIJI-Image J	NIH	RRID:SCR_002285
Imaris 7.4	Bitplane	RRID:SCR_007370
BioRender	BioRender	RRID:SCR_018361
CT-FIRE (version 2.0 beta)	University of Wisconsin System	https://loci.wisc.edu/software/ctfire

References

1. Wang, L.C. *et al.* Targeting fibroblast activation protein in tumor stroma with chimeric antigen receptor T cells can inhibit tumor growth and augment host immunity without severe toxicity. *Cancer immunology research* **2**, 154-166 (2014).
2. Lo, A. *et al.* Tumor-Promoting Desmoplasia Is Disrupted by Depleting FAP-Expressing Stromal Cells. *Cancer research* **75**, 2800-2810 (2015).
3. Watanabe, K. *et al.* Pancreatic cancer therapy with combined mesothelin-redirected chimeric antigen receptor T cells and cytokine-armed oncolytic adenoviruses. *JCI Insight* **3** (2018).
4. Klampatsa, A. *et al.* Analysis and Augmentation of the Immunologic Bystander Effects of CAR T Cell Therapy in a Syngeneic Mouse Cancer Model. *Mol Ther Oncolytics* **18**, 360-371 (2020).
5. Lee, I.K. *et al.* Monitoring Therapeutic Response to Anti-Fibroblast Activation Protein (FAP) CAR T Cells using [18F]AlF-FAPI-74. *Clinical cancer research : an official journal of the American Association for Cancer Research* (2022).
