# Supplementary material for: Desmoplastic stroma restricts T cell extravasation and mediates immune exclusion and immunosuppression in solid tumors
Source: Nat Commun. 2023 Aug 22;14:5110. doi: 10.1038/s41467-023-40850-5 (PMC10444764; doi:10.1038/s41467-023-40850-5)
Supplement: Supplementary file 3 — Description of Additional Supplementary Files [file 41467_2023_40850_MOESM3_ESM.pdf]

Description of Additional Supplementary Files

Supplementary Movie 1: MigR control T cells in tumour stroma at day 7 post administration.

Supplementary Movie 2: Meo-CAR T cells in tumour stroma at day 7 post administration.

Supplementary Movie 3: FAP-CAR T cells in tumour stroma at day 4 post administration.

Supplementary Movie 4: FAP-CAR T cells in tumour boarder at day 4 post administration.

Supplementary Movie 5: FAP-CAR T cells in tumour stroma at day 7 post administration.

Supplementary Movie 6: FAP-CAR T cells in tumour nest at day 7 post administration.

Supplementary Movie 7: FAP-CAR T cells penetrating the tumour and moving to targeted cells.

Supplementary Movie 8: FAP-CAR T cells interacting with targeted cells.

Supplementary Movie 9: Tdtomato+ Meso-CAR T cells accumulating in the tumour stroma at day 1 post administration of FAP-CAR T cells.

Supplementary Movie 10: Tdtomato+ Meso-CAR T cells successfully infiltrating into the tumour nest at day 7 post administration of FAP-CAR T cells.
