# Supplementary material for: Desmoplastic stroma restricts T cell extravasation and mediates immune exclusion and immunosuppression in solid tumors
Source: Nat Commun. 2023 Aug 22;14:5110. doi: 10.1038/s41467-023-40850-5 (PMC10444764; doi:10.1038/s41467-023-40850-5)
Supplement: Supplementary file 14 — Reporting Summary [file 41467_2023_40850_MOESM14_ESM.pdf]

nature portfolio

Corresponding author(s): Ellen Puré

Last updated by author(s): Aug 13, 2023

Reporting Summary

Nature Portfolio wishes to improve the reproducibility of the work that we publish. This form provides structure for consistency and transparency in reporting. For further information on Nature Portfolio policies, see our Editorial Policies and the Editorial Policy Checklist.

Statistics

For all statistical analyses, confirm that the following items are present in the figure legend, table legend, main text, or Methods section.

n/a	Confirmed	
☐	☒	The exact sample size (n) for each experimental group/condition, given as a discrete number and unit of measurement
☐	☒	A statement on whether measurements were taken from distinct samples or whether the same sample was measured repeatedly
☐	☒	The statistical test(s) used AND whether they are one- or two-sided<br>*Only common tests should be described solely by name; describe more complex techniques in the Methods section.*
☒	☐	A description of all covariates tested
☐	☒	A description of any assumptions or corrections, such as tests of normality and adjustment for multiple comparisons
☐	☒	A full description of the statistical parameters including central tendency (e.g. means) or other basic estimates (e.g. regression coefficient) AND variation (e.g. standard deviation) or associated estimates of uncertainty (e.g. confidence intervals)
☒	☐	For null hypothesis testing, the test statistic (e.g. F, t, r) with confidence intervals, effect sizes, degrees of freedom and P value noted<br>*Give P values as exact values whenever suitable.*
☒	☐	For Bayesian analysis, information on the choice of priors and Markov chain Monte Carlo settings
☒	☐	For hierarchical and complex designs, identification of the appropriate level for tests and full reporting of outcomes
☒	☐	Estimates of effect sizes (e.g. Cohen's d, Pearson's r), indicating how they were calculated

Our web collection on statistics for biologists contains articles on many of the points above.

Software and code

Policy information about availability of computer code

Data collection	Vevo 2100 Micro-Ultrasound Software (version 1.4), FACS Diva (version 6.0), Flowjo V10, LAS X software (Leica), NIS-Elements Advanced Research software (Nikon, version 4.50)
Data analysis	Small animal ultrasound data were analyzed by the Vevo 2100 Micro-Ultrasound Software (Visual Sonics). Two-photon microscopy imaging data were analyzed using FIJI-Image J and Imaris 7.4. Fluorescent microscopy imaging data were analyzed using LFIJI-Image J. SHG data was analyzed by using CT-FIRE (version 2.0 beta). All statistical calculations were performed using GraphPad Prism 9.0 (GraphPad Software Inc.) Flow cytometry results were analyzed by FACS and FlowJo v10.

For manuscripts utilizing custom algorithms or software that are central to the research but not yet described in published literature, software must be made available to editors and reviewers. We strongly encourage code deposition in a community repository (e.g. GitHub). See the Nature Portfolio guidelines for submitting code & software for further information.

Data

Policy information about availability of data

All manuscripts must include a data availability statement. This statement should provide the following information, where applicable:

- Accession codes, unique identifiers, or web links for publicly available datasets
- A description of any restrictions on data availability
- For clinical datasets or third party data, please ensure that the statement adheres to our policy

The processed multiplex IF and 2-photon microscopy images and movies are available at Dryad [https://datadryad.org/stash/share/-WPXeg6_fw-CX-iSeRi3-

BULdis7d0FbETaoNzMXx0Q]. The remaining data are available within the Article, Supplementary Information or Source Data file. Source data are provided with this paper.

Human research participants

Policy information about studies involving human research participants and Sex and Gender in Research.

Reporting on sex and gender	N/A
Population characteristics	N/A
Recruitment	N/A
Ethics oversight	N/A

Note that full information on the approval of the study protocol must also be provided in the manuscript.

Field-specific reporting

Please select the one below that is the best fit for your research. If you are not sure, read the appropriate sections before making your selection.

☒ Life sciences ☐ Behavioural & social sciences ☐ Ecological, evolutionary & environmental sciences

For a reference copy of the document with all sections, see nature.com/documents/nr-reporting-summary-flat.pdf

Life sciences study design

All studies must disclose on these points even when the disclosure is negative.

Sample size	No effect size was predetermined, but sample sizes employed in this study are consistent with previously published works (LWang, Y., Zhou, SK., Wang, Y. et al. Nat Commun 14, 1993 (2023); or KLi, N., Quan, A., Li, D. et al. Nat Commun 14, 1986 (2023).). For example, in vitro studies were repeated at least three times independently and in the in vivo experiments with at least 5 mice per group were performed.
Data exclusions	No data was excluded from the analysis.
Replication	Experiments were repeated at least three independent experiments with similar results. All experiments were reproduced to reliably support conclusions stated in the manuscript.
Randomization	For the transplant models, tumor cells were inoculated into female mice aged 8-10 weeks of similar weight, randomized before tumor inoculation. Tumor volume was calculated as width × width × length × 0.5 and randomly assigned to the various treatment groups. KPC mice were enrolled based on the size of spontaneously arising tumors rather than age or weight. KPC mice were monitored by ultrasound and were randomly assigned to the various treatment groups when the tumor volume reached 50-100 mm3.
Blinding	Investigators were blinded to group allocation during experiments. Investigators performing tumor measurements were blinded to treatment procedures during data collection and analysis.

Reporting for specific materials, systems and methods

We require information from authors about some types of materials, experimental systems and methods used in many studies. Here, indicate whether each material, system or method listed is relevant to your study. If you are not sure if a list item applies to your research, read the appropriate section before selecting a response.

Materials & experimental systems

n/a	Involved in the study	
☐	☒	Antibodies
☐	☒	Eukaryotic cell lines
☒	☐	Palaeontology and archaeology
☐	☒	Animals and other organisms
☒	☐	Clinical data
☒	☐	Dual use research of concern

Methods

n/a	Involved in the study	
☒	☐	ChIP-seq
☐	☒	Flow cytometry
☒	☐	MRI-based neuroimaging

Antibodies

Antibodies used	Alexa Fluor® 700 anti-mouse CD45 (1:100,Cat#: 157616), Alexa Fluor® 700 anti-mouse CD45.2 (1:100,Cat#: 109822), Brilliant Violet 605™ anti-mouse CD45.1 Antibody (1:100, Cat#: 110738), APC/Cyanine7 anti-mouse CD45.1 (1:100, Cat#: 110716), PE/Cyanine5 anti-mouse CD3ε (1:100, Cat#: 100310), APC/Cyanine7 anti-mouse CD4 (1:100, Cat#: 100414), Brilliant Violet 421™ anti-mouse CD4 (1:100, Cat#: 100438), Brilliant Violet 785™ anti-mouse CD279 (PD-1, 1:100,Cat#: 135225), Brilliant Violet 650™ anti-mouse/human Ki-67 (1:100,Cat#: 151215), PE anti-mouse CD69 (1:100, Cat#: 104508), Brilliant Violet 421™ anti-mouse LAP (TGF-β1, 1:100, Cat#: 141408), PerCP/Cyanine5.5 anti-mouse LAP (TGF-β1, 1:100,Cat#: 141410), Alexa Fluor® 647 anti-mouse FOXP3 (1:100, Cat#: 126408), Brilliant Violet 605™ anti-mouse Ly-6C (1:100, Cat#: 128036), APC anti-mouse Ly-6C (1:100, Cat#: 128016), APC/Cyanine7 anti-mouse I-A/I-E (1:100, Cat#: 107628), PE/Cyanine5 anti-mouse I-A/I-E (1:100, Cat#: 107612), PE anti-mouse Ly-6G (1:100, Cat#: 127608), Brilliant Violet 650™ anti-mouse CD11c (1:100, Cat#: 1173390), Brilliant Violet 605™ anti-mouse CD103 (1:100, Cat#: 121433), Brilliant Violet 650™ anti-mouse CD206 (MMR, 1:100, Cat#: 141723), PE/Cyanine5 anti-mouse CD19 (1:100, Cat#: 115510), PE/Cyanine7 anti-mouse NK-1.1 (1:100, Cat#: 156514), APC/Cyanine7 anti-mouse CD326 (Ep-CAM,1:100, Cat#: 118218), Alexa Fluor® 647 anti-mouse CD326 (Ep-CAM, 1:100,Cat#: 118212), PE/Cyanine7 anti-mouse CD326 (Ep-CAM, 1:100, Cat#: 118216), Brilliant Violet 785™ anti-mouse CD90.2 (Thy-1.2, 1:100, Cat#: 105331), PE anti-mouse CD90.2 (Thy-1.2, 1:100, Cat#: 105308), Alexa Fluor® 647 anti-mouse CD90.2 (Thy1.2, 1:100, Cat#: 105318), Alexa Fluor® 647 anti-mouse CD31 (1:100, Cat#: 102416), PE anti-mouse IFN-γ (1:100, Cat#: 505808), Brilliant Violet 711™ anti-mouse IFN-γ (1:100, Cat#: 5058360), APC anti-mouse TNF- α (1:100, Cat#: 506308), Brilliant Violet 650™ anti-mouse TNF-α (1:100, Cat#: 506333), PerCP/Cyanine5.5 anti-human/mouse Granzyme B Recombinant Antibody (1:100, Cat#: 372212), PE anti-human/mouse Granzyme B Recombinant Antibody (1:100, Cat#: 372208) were purchased from Biolegend. BUV805 Mouse Anti-Mouse CD45.1 (1:100, Cat#: 612900), BUV805 Rat Anti-Mouse CD4 (1:100, Cat#: 612900), BUV395 Mouse Anti-Mouse CD366 (TIM-3, 1:100, Cat#: 747620), PerCP-Cy™5.5 Rat Anti-CD11b (1:100, Cat#: 550993), BUV395 Rat Anti-Mouse Ly-6G (1:100, Cat#: 563978), BUV805 Hamster Anti-Mouse CD11c (1:100, Cat#: 749090), BV421 Hamster Anti-Mouse CD11c (1:100, Cat#: 562782), BV711 Rat Anti-Mouse F4/80 (1:100, Cat#: 565612) were purchased from BD Biosciences. PE-Cyanine7 CD8a Monoclonal (1:100, Cat#: 25-0081-82) were purchased from eBioscience. Primary antibodies were: Recombinant Anti-Fibroblast activation protein, alpha antibody, 1:1000, Abcam, Cat#: ab207178; Goat polyclonal Anti-GFP, 1:1000, Abcam, Cat#: ab6673; Rabbit polyclonal Anti-CD3,1:1000, Abcam, Cat#: ab5690; Recombinant Anti-CD8 alpha,1:1000, Abcam, Cat#: ab217344; Recombinant Anti-CD4 ,1:1000, Abcam, Cat#: ab183685; Recombinant Anti-FOXP3,1:1000, Abcam, Cat#: ab215206; Recombinant Anti-F4/80, 1:1000, Abcam, Cat#: ab111101; Recombinant Anti-CD103, 1:1000, Abcam, Cat#: ab224202; Anti-Ly6g+Ly6c (Gr-1), 1:1000, Abcam, Cat#: ab25377; Recombinant Anti-Ki67, 1:1000, Abcam, Cat#: ab16667; Recombinant Anti-Cytokeratin 19, 1:1000, Abcam, Cat#: ab52625; Recombinant Anti-Mesothelin , 1:1000, Abcam, Cat#: ab187063; Mesothelin Monoclonal, MSLN, 1:1000, NeoBiotechologies, Cat#: 10232-MSM1-P1; Monoclonal Rat anti-Mouse MSLN/Mesothelin , 1:1000, LSBio, Cat#: LS-C179484-100; TGF beta 1 Polyclonal , 1:1000, Bioss, Cat#: BS-0086R; Mouse/Rat CD31/PECAM-1 , 1:1000, R&D Systems, Cat#: AF3628; Mouse PDGFR alpha, 1:1000, R&D Systems ,Cat#: AF1062; Mouse Podoplanin, 1:1000, R&D Systems Cat#: AF3244; Mouse/Rat IFN-gamma Antibody , 1:1000, R&D Systems, Cat#: AF-585-NA; Mouse MMR/CD206, 1:1000, R&D Systems, Cat#: AF2535; Mouse PD-1 Antibody, 1:1000 , R&D Systems, Cat#: AF1021; Rabbit Polyclonal Anti-RFP , 1:1000, Rockland, Cat#: 600-401-379; Collagen Hybridizing Peptide, Biotin Conjugate (B-CHP) , 1:1000, 3Helix, Cat#: BIO300; Rabbit Polyclonal Anti-Fibronectin , 1:1000, Sigma-Aldrich, Cat#: F3648; Rabbit Cleaved Caspase-3 , 1:1000, Cell Signaling Technology, Cat#: 9664. Secondary antibodies were: Alexa Fluor™ Plus 488 Donkey anti-Rabbit (1:1000, Invitrogen, Cat#: A32790), Alexa Fluor™ Plus 555 Donkey anti-Rabbit (1:1000, Invitrogen, Cat#: A32794), Alexa Fluor™ Plus 647 Donkey anti-Rabbit (1:1000, Invitrogen, Cat#: A32795), Alexa Fluor™ Plus 488 Donkey anti-Goat (1:1000, Invitrogen, Cat#: A32814), Alexa Fluor™ Plus 555 Donkey anti-Goat (1:1000, Invitrogen, Cat#: A32816), Alexa Fluor™ Plus 647 Donkey anti-Goat (1:1000, Invitrogen, Cat#: A32849), DyLight™ 488 Donkey anti-Rat (1:1000, Invitrogen, Cat#: SA5-10026), DyLight™ 550 Donkey anti-Rat (1:1000, Invitrogen, Cat#: SA5-10027), DyLight™ 650 Donkey anti-Rat (1:1000, Invitrogen, Cat#: SA5-10029), DyLight™ 488 Donkey anti-Mouse (1:1000, Invitrogen, Cat#: SA5-10166), DyLight™ 550 Donkey anti-Mouse (1:1000, Invitrogen, Cat#: SA5-10167), DyLight™ 560 Donkey anti-Mouse (1:1000, Invitrogen, Cat#: SA5-10169). InVivoMAb anti-mouse PD1 (Cat#: BE0146) was purchased from BioXCell. CD16/CD32 Monoclonal Antibody (Cat#: 14-0161-82) eBioscience. APC AffiniPure F(ab')$_2$ Fragment Goat Anti-Mouse IgG, F(ab')$_2$ fragment specific (1:1000, Cat#: 115-136-072), APC AffiniPure F(ab')$_2$ Fragment Goat Anti-Human IgG, F(ab')$_2$ fragment specific (1:1000, Cat#: 109-136-097) were purchased from Jackson ImmunoResearch. Mouse monoclonal Anti-Actin, α-Smooth Muscle - Cy3™ (1:1000, Cat#: C6198) were purchased from Sigma-Aldrich, Alexa Fluor™ 488 Pan-Cytokeratin Monoclonal (1:100, Clone AE1/AE3, Cat#: 53-9003-82) were purchased from eBioscience. InVivoMAb anti-mouse PD1 (RMP1-14, Cat#: BE0146) was purchased from BioXCell (5 mg/kg twice a week).
Validation	All antibodies used in this study were commercial and validated by the manufacturer. Species and application validations and citations for primary antibodies can be found from the manufacturer's websites.

Eukaryotic cell lines

Policy information about cell lines and Sex and Gender in Research

Cell line source(s)	Human CCD-19 lung fibroblast (CCD19-Lu), human AsPC-1 and Capan-2 PDAC tumor cells, human 293T cells and Phoenix-ECO packaging cells were purchased from ATCC. Mesothelin-positive 4662 cells were established from the fully backcrossed C57BL/6J KPC PDAC mouse model and kindly provided by Dr. Robert H. Vonderheide (University of Pennsylvania). 3T3.mFAP.GFP/Luc expressing high level of FAP and 3T3.GFP/Luc without FAP expression cells were generated by transduction with the lentivirus of firefly luciferase as previously described. All of the cell lines were tested negative for mycoplasma in University of Pennsylvania cell center.
Authentication	A short tandem repeat DNA profiling method was used to authenticate the cell lines and the results were compared with reference database. There is no mycoplasma contamination in the above cell lines.
Mycoplasma contamination	All cell lines were tested for mycoplasma contamination. No mycoplasma contamination was found.
Commonly misidentified lines (See ICLAC register)	These cell lines we used were not listed in commonly misidentified lines in ICLAC Register.

Animals and other research organisms

Policy information about studies involving animals; ARRIVE guidelines recommended for reporting animal research, and Sex and Gender in Research

Laboratory animals	C57BL/6J (CD45.1 donor and CD45.2 recipient), NOD/SCID/IL2-receptor γ chain knockout (NSG) and B6.129(Cg)-Gt(ROSA)26Sortm4 (ACTB-tdTomato,-EGFP)Luo/J (mT/mG) mice were purchased from Jackson Laboratory. Fully backcrossed KrasG12D;Trp53R172H;Pdx-1-Cre (KPC) C57BL/6 mice were bred and and housed in a specific-pathogen-free animal facility at ambient temperature (22 ± 2 °C), air humidity 40%–70% and 12-h dark/12-h light cycle and had free access to water and chow. Animal health status was routinely checked by qualified veterinarians.
Wild animals	No wild animal was involved in this study.
Reporting on sex	For the transplant models, tumor cells were inoculated into female mice aged 8-10 weeks of similar weight, randomized before tumor inoculation. KPC mice were enrolled based on the size of spontaneously arising tumors rather than age or weight. All animal experiments were performed in the same well-controlled pathogen-free facility and fed ad libitum. Since the cell line (4662) for the syngeneic transplant model was derived from a tumor from a female KPC mouse, only female mice hosts were utilized. To be consistent, only female mice were used for the xenograft models. However, male and female mice were included in the studies of the genetically engineered autochthonous KPC model. As based on two-side Student's t tests, tumor growth was comparable in male and female KPC mice, the data from both sexes were analyzed together.
Field-collected samples	The study did not involve samples collected from the field.
Ethics oversight	All animal experiments were approved by the Institutional Animal Care and Use Committee (IACUC) of the University of Pennsylvania (Animal Welfare Assurance no.: D16-00045 [A3079-07], Protocol no.: 805003 and 805004) and were carried out in accordance with the guidelines.

Note that full information on the approval of the study protocol must also be provided in the manuscript.

Flow Cytometry

Plots

Confirm that:

- [x] The axis labels state the marker and fluorochrome used (e.g. CD4-FITC).
- [x] The axis scales are clearly visible. Include numbers along axes only for bottom left plot of group (a 'group' is an analysis of identical markers).
- [x] All plots are contour plots with outliers or pseudocolor plots.
- [x] A numerical value for number of cells or percentage (with statistics) is provided.

Methodology

Sample preparation	Tumor tissue was dissected and digested with 2.5 mg/mL Collagenase type 2 (Worthington) plus 0.25 mg/mL DNase I (Roche) in RPMI-1640 for 30 minutes with intermittent shaking at 37°C. Digestion mixture was passed through 70 µm followed by 40 µm cell strainers (FALCON) to prepare single cell suspension and washed with stain buffer (BD Biosciences). The spleens were mashed and passed through 40 µm cell strainer (FALCON). Red blood cells (RBC) were removed using RBC lysis buffer (BD Biosciences). Blood was collected through retro-orbital bleeding and RBC was removed using the same RBC lysis buffer. After washing with PBS, cells were collected. Single-cell suspensions were obtained and stained with antibodies according to the manufacturer's protocols, and then analyzed by flow cytometry.
Instrument	LSRFortessa, Symohony A3 Lite (BD Biosciences)
Software	FACS Diva v6.0 and FlowJo V10
Cell population abundance	The absolute cells at least 100000 were analyzed for fluorescent intensity in the defined gate.
Gating strategy	Briefly, single cells were selected by FSC and SSC plots. Live cells were selected as defined by Live Dead Stain-negativity. Detailed gating strategies were provided in the Extended Data Figs. 4, 6, 8 and 9. For example, immune cells were gated by CD45+ cells. CAR-T cells were gated by CD45.1+CD3+ cells. Endogenous T cells were gated by CD45.1-CD3+ cells. Myeloid cells were gated by CD11b+ cell, and macrophages were gated by F4/80+ cells. Epithelial cells were gated by CD45-/CD31-/CD326+ cells.

- [x] Tick this box to confirm that a figure exemplifying the gating strategy is provided in the Supplementary Information.
